# CHILDREN’S HEALTH: Gene Variants May Predict Lung Health

**DOI:** 10.1289/ehp.117-a243

**Published:** 2009-06

**Authors:** Bob Weinhold

Genetic research is beginning to provide potential insights on why some people are more vulnerable than others to various pollutants. Now a few more pieces can be added to the extraordinarily complex puzzle. In the April 2009 *American Journal of Respiratory and Critical Care Medicine*, researchers from the University of Southern California report that several inherited glutathione *S*−transferase (GST) gene variants were associated with lung function growth during adolescence, and one variant helped predict poor lung function in children whose mothers had smoked during pregnancy.

GST genes are expressed in the lungs, among other organs, and may influence the development of normal lung function. This study focused on a class of GST genes called *GSTM*s. The best studied of these is *GSTM1*, which has been implicated in lung cancer, asthma, and other respiratory diseases in children and adults. To find out more about other variants, the authors looked at *GSTM2*, *GSTM3*, *GSTM4*, and *GSTM5*. They analyzed the genotypes in conjuction with eight years’ worth of lung function and genotyping data from 2,108 Southern California schoolchildren, starting around age 10 years.

For *GSTM2*, two common haplotypes (patterns of genetic variation) were observed. One haplotype found in 28% of non−Hispanic whites and 44% of Hispanic whites was associated with better lung development than other haplotypes, whereas a second haplotype found in 35% of non−Hispanic whites and 30% of Hispanic whites was associated with significantly less lung function growth over time. Deficits in measures of lung function growth were twice as big in children with two copies of the haplotype versus those with one copy, and the deficits associated with this haplotype were greater among children of mothers who smoked during pregnancy.

The presence of a *GSTM4* haplotype found in 22% of non−Hispanic whites and 16% of Hispanic whites was associated with significantly reduced lung function growth relative to children with other haplotypes. As with the *GSTM2* variant above, deficits in lung function growth were doubled among children with 2 copies of the haplotype. A *GSTM3* haplotype found in about 7% of the children was associated with a deficit in one of three measures of lung function growth.

The authors believe this to be the first study to report evidence of an interaction between *GSTM2* variants and prenatal tobacco smoke exposure on lung function growth. They noted that *GSTM* genes are expressed in fetal tissue at relatively low levels, and GSTs and other phase II (detoxification) enzymes are relatively inactive in fetal tissue compared with phase I enzymes (which activate toxic metabolites and carcinogens). “This suggests that fetuses are much more susceptible to environmental exposures than adults,” they wrote. “Genetic polymorphisms in phase II enzymes that further inhibit enzyme activity may exacerbate this susceptibility.” In addition, respiratory health and development in childhood is known to play an important role in adult respiratory health.

This study “points out again that the genetic makeup of a population may be very important when considering health risks from environmental exposures,” says principal investigator Frank Gilliland, who directs the NIEHS−supported Southern California Environmental Health Sciences Center at the University of Southern California. His team speculates that *GSTM*−influenced failure to detoxify reactive oxygen species reduces lung protection and triggers an inflammatory cascade, bronchial constriction, and airway hyperresponsiveness, producing asthma−like symptoms and impaired lung development.

However, more information would be needed before public health officials could set or adjust regulatory standards based on variation in vulnerability among subpopulations. And even with such additional data, Medea Imboden, a molecular epidemiologist at the University of Zürich, cautions against overemphasizing the GST gene family. “You always have doubts about the functional relevance of the variants,” says Imboden. “If you think of all the genes not yet known to contribute to respiratory health and bodily defense systems against air pollutants, GSTs might turn out to play a minor role.” But for now, she adds, “they are good candidates.”

